# Robust Range Estimation with a Monocular Camera for Vision-Based Forward Collision Warning System

**DOI:** 10.1155/2014/923632

**Published:** 2014-01-16

**Authors:** Ki-Yeong Park, Sun-Young Hwang

**Affiliations:** Department of Electronic Engineering, Sogang University, CPO. Box 1142, Seoul 100-611, Republic of Korea

## Abstract

We propose a range estimation method for vision-based forward collision warning systems with a monocular camera. To solve the problem of variation of camera pitch angle due to vehicle motion and road inclination, the proposed method estimates virtual horizon from size and position of vehicles in captured image at run-time. The proposed method provides robust results even when road inclination varies continuously on hilly roads or lane markings are not seen on crowded roads. For experiments, a vision-based forward collision warning system has been implemented and the proposed method is evaluated with video clips recorded in highway and urban traffic environments. Virtual horizons estimated by the proposed method are compared with horizons manually identified, and estimated ranges are compared with measured ranges. Experimental results confirm that the proposed method provides robust results both in highway and in urban traffic environments.

## 1. Introduction

As number of vehicles increases, driving safety has become an important issue. Since a considerable number of traffic accidents are caused by driver inattention, driver assistance systems which detect imminent collision and provide timely warning for drivers can significantly reduce the number and severity of these accidents [1]. Forward collision warning (FCW) system detects vehicles ahead and issues warnings in advance to drivers for avoiding or mitigating the harm caused by rear-end crashes [2–6]. FCW system identifies a target vehicle within trajectory of subject vehicle and determines range to the target vehicle. Time-to-collision (TTC) is computed using the range, and collision warning is issued when TTC is lower than a certain threshold [7, 8].

To issue timely collision warning, range to target vehicle has to be determined accurately. Radar sensors have been commonly used for this purpose. They can detect objects over long range even under poor illumination conditions [1, 9, 10]. Due to their cost and limited angular accuracy, vision-based FCW systems which use a low-cost image sensor have been popularly investigated by many researchers [2, 5, 6, 11, 12]. Contrary to radar sensors, image sensors do not provide range information. Range should be estimated from information on size and/or position of vehicles in image [6, 11, 13–18]. Width of a vehicle in image is inversely proportional to range to the vehicle, and range can be estimated from vehicle width in image. However, range estimation from vehicle width without prior knowledge of vehicle real width may introduce significant error, as vehicle real width varies from 1.4 m to 2.6 m. Distance between vehicle bottom and horizon in image is inversely proportional to range to the vehicle. Range can be estimated accurately from vehicle bottom position in image, if horizon is located accurately. As position of horizon varies due to vehicle motion and road inclination, it has to be located at run-time. Position of horizon can be located by analyzing lane markings [6, 13]. However, this method cannot be appropriately used when road inclination varies continuously on hilly roads or lane markings are not seen on crowded roads. Moreover, horizon estimated by analyzing lane markings in close proximity may result in unbounded range error, especially when vehicles are on a hill.

In this paper, we propose a robust range estimation method which provides range information even when road inclination varies continuously or lane markings are not seen. The proposed method estimates virtual horizon from information on size and position of vehicles and calculates range from vehicle position with the estimated virtual horizon. The rest of this paper is organized as follows. In Section 2, we present a brief introduction of vision-based FCW system and describe conventional vision-based range estimation methods. The proposed range estimation method is presented in Section 3. Experimental results are presented in Section 4 and the conclusions are drawn in Section 5.

## 2. Backgrounds

### 2.1. Vision-Based FCW System

In this section, a brief introduction of vision-based FCW system is presented, which adopts object detection algorithm to determine size and position of vehicles in image. Object detection is to distinguish a class of objects which have large intraclass variability such as faces, pedestrians, and vehicles from cluttered background [19, 20]. Many vision-based vehicle detection systems have been designed only for highway traffic where lanes are usually well marked and built with slowly changing curvature [2, 21, 22]. Whereas vehicles appear quite separated from background in highway traffic environment, vehicles have to be distinguished from more cluttered background in urban traffic environment [23]. Most FCW systems adopt object detection algorithm for this purpose [11, 24–26].

**Figure 1:**
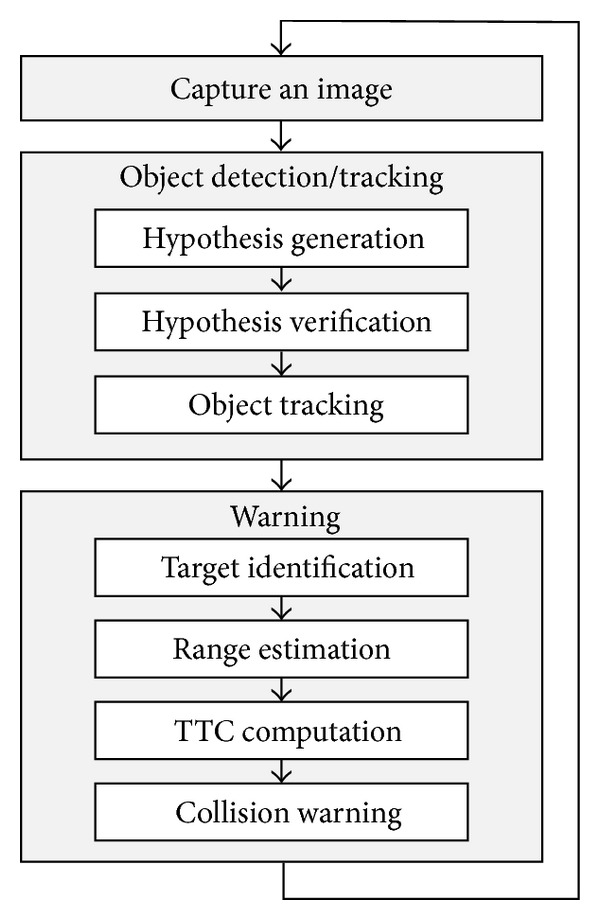
Vision-based FCW system.

Figure 1 shows the flow of tasks performed by a vision-based FCW system which adopts object detection algorithm. It consists of two stages: object detection/tracking stage and warning stage. In object detection/tracking stage, size and position of vehicles in captured image are determined. Most object detection algorithms consist of hypothesis generation and hypothesis verification phases [27]. Presence of vehicles is hypothesized in hypothesis generation phase by using prior knowledge about vehicle such as color, texture, symmetry, corners, and horizontal/vertical edges [27]. In hypothesis verification phase, the presence of vehicles is verified with a classifier which distinguishes vehicles from background by computing a series of features. Sun et al. employed Haar wavelet decomposition for feature extraction and support vector machines (SVM) for classification [24]. Cui et al. employed Haar-like features for feature extraction and AdaBoost for classification [11]. Accuracy of object detection can be improved by combining it with tracking mechanism. Presence of vehicles in future frames is hypothesized by using detection result of current frame, and false detections are reduced by validating detection results with past history. Betke et al. used a refined search within tracking window to validate detections [28]. Cui et al. employed simplified Lucas-Kanade algorithm to remove false detections [11].

In warning stage, TTC is computed for a target vehicle and collision warning is issued [7, 8]. Trajectory of subject vehicle is predicted by using road lane information and vehicle signals such as steering angle, yaw, and speed [5]. The nearest vehicle within the trajectory of subject vehicle is identified as a target vehicle. To issue collision warning, range to the target vehicle is determined and TTC is computed using the range. Collision warning is triggered when TTC is lower than a certain threshold, which is in the range of 2.0~2.4 sec according to NHTSA NCAP Forward Collision Warning System Confirmation Test [29].

### 2.2. Range Estimation with a Monocular Camera

In this section, conventional range estimation methods used in vision-based systems with a monocular camera are described. As image sensors do not provide range information, range should be estimated from information on size and/or position of vehicle in image by using the pinhole camera geometry [30].

#### 2.2.1. Range Estimation Using Size Information

Width of a vehicle in image is proportional to real width of the vehicle according to the pinhole camera geometry. If real width of a vehicle is known, range *d* to the vehicle can be calculated as in the following:

(1) $$\begin{array}{c} d=F_{c}\cdot \frac{W_{a}}{w_{a}}, \end{array}$$

where *F*_*c*_ is focal length of camera and *w*_*a*_ and *W*_*a*_ are vehicle width in image and vehicle real width, respectively. Vehicle real width varies from 1.4 m to 2.6 m. Applying this formula for range estimation without prior knowledge of vehicle real width may introduce significant error, which can be as much as 30%, if a fixed width (e.g., *W*_*a*_ = 1.82 m) is used. It is not accurate enough for computing TTC, but it can be used as sanity check [13].

#### 2.2.2. Range Estimation Using Position Information

**Figure 2:**
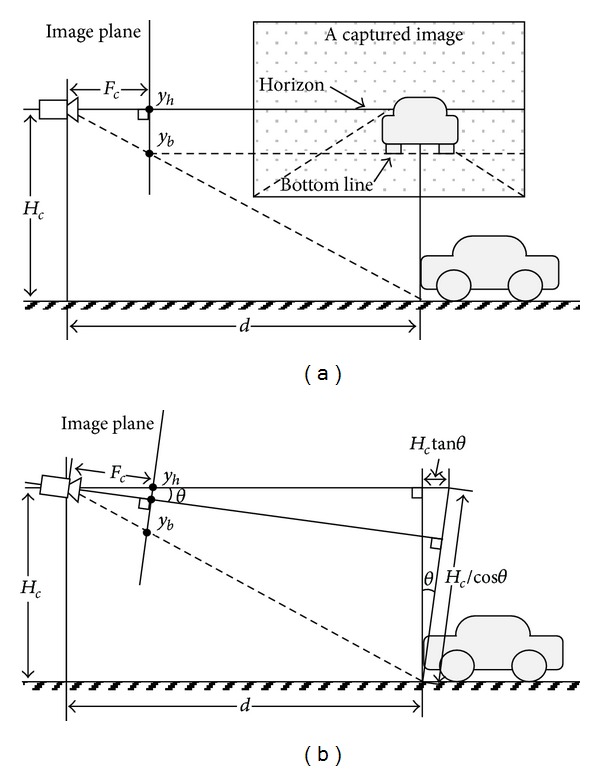
Range estimation from vehicle position in image by using the pinhole camera geometry. (a) Camera pitch angle is zero; (b) camera pitch angle is nonzero.

With an assumption that both roll and yaw angles are zero, range can be estimated from vehicle position in image by using the pinhole camera geometry as shown in Figure 2. Range estimation when camera pitch angle is zero and nonzero is illustrated in Figures 2(a) and 2(b), respectively. Let contacting line between vehicle bottom and road surface in image be *bottom line* and let horizontal line passing through vanishing point of road lanes be *horizon*. Bottom line of a vehicle approaches to horizon as the vehicle goes far away from camera. Horizon will pass through the centre of image when optical axis of camera is parallel to road surface. It moves upward or downward depending on camera pitch angle.

Distance between bottom line of a vehicle and horizon is inversely proportional to range to the vehicle. When camera pitch angle is negligibly small, range *d* to vehicle can be calculated as in the following:

(2) $$\begin{array}{c} d=\frac{F_{c}\cdot H_{c}}{y_{b}-y_{h}}, \end{array}$$

where *F*_*c*_ is camera focal length, *H*_*c*_ is camera height, and *y*_*b*_ and *y*_*h*_ are vertical coordinates of vehicle bottom line and horizon, respectively. When camera pitch angle *θ* is considerably large, range has to be calculated as in the following:

(3) $$\begin{array}{c} d=\frac{1}{\cos^{2}\;\theta }\cdot \frac{F_{c}\cdot H_{c}}{y_{b}-y_{h}}-H_{c}\tan\theta . \end{array}$$

If *θ* is small, (2) can be used instead of (3), since range error resulting by small *θ* is negligible. For example, when camera height *H*_*c*_ is 1.3 m and *θ* is 10°, 1/cos^2^ *θ* is 1.03 and the second term in (3) becomes 0.23 m. However, when *θ* is nonzero, horizon does not pass through the centre of image anymore, and its position has to be determined.

Small variations in horizon position may result in large range error, since the denominator term *y*_*b*_ − *y*_*h*_ in (2) becomes very small as vehicle goes far away from camera. In highway traffic environment where horizon varies in a small range, range can be calculated with a fixed horizon determined by camera calibration. In urban traffic environment where horizon can vary much due to vehicle motion and road inclination, it should be located at run-time. Horizon can be determined by analyzing lane markings [6, 13]. However, this method cannot be appropriately used when road inclination varies continuously or lane markings are not seen.

## 3. Proposed Range Estimation Method

In this section, we propose a robust range estimation method which provides range information even when road inclination varies continuously or lane markings are not seen. The proposed range estimation method determines *virtual horizon* only from size and position of vehicles in image and calculates range with the estimated virtual horizon.

### 3.1. Virtual Horizon Estimation

If real width *W*_*a*_ of a vehicle is known and both size and position of the vehicle in image are given, vertical coordinate *y*_*h*_ of horizon can be determined from (1) and (2) and presented in the following:

(4) $$\begin{array}{c} y_{h}=y_{b}-H_{c}\cdot \frac{w_{a}}{W_{a}}, \end{array}$$

where *H*_*c*_ is camera height which is constant and *y*_*b*_ and *w*_*a*_ are vehicle bottom line position and vehicle width in image, respectively, which are obtained in object detection stage. Vehicle real width *W*_*a*_ can be represented as $\bar{W_{a}}+\Delta W_{a}$, where $\bar{W_{a}}$ is average real width of vehicles and Δ*W*_*a*_ is difference between real width of a vehicle and the average real width. If sufficiently many vehicles are detected, ∑(Δ*W*_*a*_) converges to zero and can be ignored. As a result, horizon can be determined only from the information on position and width of detected vehicles with a fixed average real width (e.g., $\bar{W_{a}}=1.82\;$m).

When several vehicles are detected in object detection stage, average horizon $\bar{y_{h}}$ can be determined from average of vehicle positions and average of vehicle widths with a fixed real width as in the following:

(5) yh¯=1N·∑i=1N(yb,i−Hc·wa,iWa¯+ΔWa,i)≃yb¯−Hc·wa¯Wa¯,

where *N* is number of detected vehicles and *y*_*b*,*i*_ and *w*_*a*,*i*_ are position and width of a detected vehicle, respectively. $\bar{y_{b}}$ and $\bar{w_{a}}$ are average position and average width of detected vehicles, respectively. Δ*W*_*a*,*i*_ is difference between real width of a detected vehicle and a fixed average real width $\bar{W_{a}}$ and is ignored in (5).

Estimated horizon position can fluctuate due to occurrence of false detections and insufficient number of detections, as well as pitch motion of subject vehicle such as vibration and acceleration. Fluctuation of horizon due to pitch motion of subject vehicle can be ignored as both vehicle position and horizon in image are influenced at the same time by the pitch motion. Range error due to pitch angle itself is negligible if pitch angle is small as we described in Section 2.2.2. However, fluctuation of horizon due to false detections and insufficient number of detections need to be removed. As road inclination changes slowly in most cases, fluctuation of horizon can be reduced by reflecting the information on previously estimated horizon. *Virtual horizon* at image frame *t* can be estimated as in the following:

(6) $$\begin{array}{c} y_{h}\left(t\right)=\gamma \cdot \bar{y_{h}}\left(t\right)+\left(1-\gamma \right)\cdot y_{h}\left(t-1\right),\;0<\gamma \leq 1, \end{array}$$

where *y*_*h*_(*t*) and *y*_*h*_(*t* − 1) are virtual horizons estimated at image frames *t* and *t* − 1, respectively, and $\bar{y_{h}}(t)$ is average horizon calculated at image frame *t* obtained by applying (5). *γ* is a constant experimentally determined. When *t* = 0, a default horizon position is used for *y*_*h*_(*t* − 1). Once virtual horizon is determined, range can be calculated from vehicle position with the virtual horizon by (2).

False detections can be reduced by restricting width of detected vehicles in object detection stage. Applying virtual horizon *y*_*h*_(*t* − 1) estimated at previous image frame to (4), min/max width of a vehicle at position *y*_*b*_ in image can be restricted as in the following:

(7) $$\begin{array}{c} \frac{y_{b}-y_{h}\left(t-1\right)}{H_{c}}\cdot W_{a,\min}\leq w_{a}\leq \frac{y_{b}-y_{h}\left(t-1\right)}{H_{c}}\cdot W_{a,\max}, \end{array}$$

where *W*_*a*,min_/*W*_*a*,max_ is min/max of vehicle real width (e.g., *W*_*a*,min_ = 1.4 m, *W*_*a*,max_ = 2.6 m). A detected vehicle whose width in image is out of these bounds should be regarded as a false detection.

### 3.2. Accuracy Consideration

Range estimation from vehicle width in image can provide accurate range information only when real width of the vehicle is given, and range error can be as much as 30%, if a fixed width is used instead of real width of the vehicle as we described in Section 2.2.1. On the other hand, range estimation from vehicle position in image can provide accurate range information only when horizon is located accurately. Small variation in position of horizon may result in large range error. Horizon always has to be located above vehicle bottom lines. Range cannot be determined if vehicle is located above horizon. However, horizon estimated by analyzing lane markings in close proximity as in conventional method may be located even below vehicle bottom lines, especially when vehicles are on a hill.

In the proposed method, virtual horizon is always located above vehicle bottom lines, and range error is bounded to a limit in a condition that both size and position of detected vehicles are accurate, since the virtual horizon is also estimated from vehicle positions. In case only one vehicle is detected, the proposed method in (5) turns to the range estimation using only size information in (1). Especially when width of the only detected vehicle is very small or very large, range error may amount to as much as 30%, which is the upper limit of range error with the proposed method.

## 4. Experimental Results

**Figure 3:**
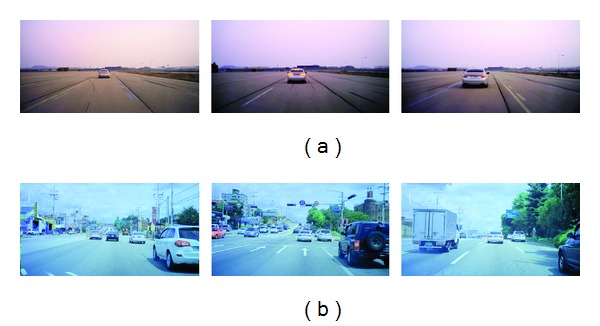
Example of captured image frames from test video clips. (a) Highway traffic environment; (b) urban traffic environment.

For experiments, a vision-based FCW system has been implemented, which employs an object detection algorithm based on Haar-like feature and AdaBoost [20, 25, 26]. Test video clips were recorded with a vehicle-mounted camera both in highway and in urban traffic environments. Resolution of the camera sensor is 1280 × 672 pixels and frame rate of the camera is 15 frames per second. Highway traffic video clips were recorded in a test track according to NHTSA NCAP Forward Collision Warning System Confirmation Test [29]. The confirmation test consists of three scenarios: stopped/decelerating/slower moving target vehicle. In all the scenarios, subject vehicle was moving at a constant speed. Urban traffic video clip includes a crossroad section where lane markings are not seen and a hill section where road inclination varies. Examples of captured image frames from the video clips are shown in Figure 3.

Image frames were captured from each video clip, and reference horizons were manually identified by analyzing lane markings for each captured image frame. Lengths of the highway traffic video clips are 56, 152, and 88 frames, respectively, and that of the urban traffic video clip is 884 frames. Virtual horizons estimated by the proposed method are compared with the reference horizons. In our experiments, *γ* = 0.2 is used to calculate virtual horizon in (6). Ranges measured by using differential GPS were prepared for each highway traffic video clip. Ranges estimated by using the proposed method are compared with the measured ranges and ranges estimated by using conventional methods described in Section 2.2. With the conventional method which estimates range from vehicle position, ranges are calculated both with reference horizons and a fixed horizon. A fixed horizon was determined for each clip by averaging the reference horizons. For the urban traffic video clip, ranges measured by using differential GPS were not prepared. Ranges estimated by using the proposed method are compared with ranges estimated by using the conventional methods.

### 4.1. Evaluation of Virtual Horizon Estimation

Virtual horizons estimated by using the proposed method are compared with reference horizons. Position of reference horizons varies in the range of 12 pixels (between 364 and 376) in the highway traffic video clips, and it varies in the range of 66 pixels (between 311 and 377) in the urban traffic video clip. The urban traffic video clip includes a hill section as well as a crossroad section. As a result, the variation in horizon positions is much increased in the clip.

**Figure 4:**
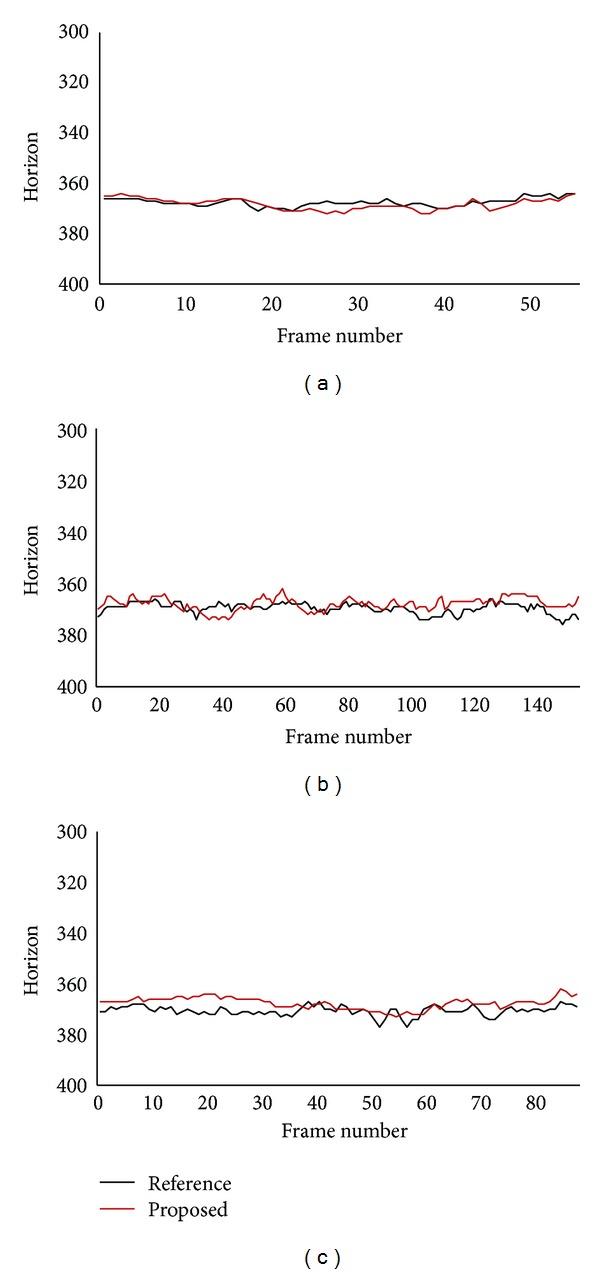
Reference horizon and virtual horizon estimated by the proposed method for highway traffic video clips. (a) Stopped target vehicle; (b) decelerating target vehicle; (c) slower moving target vehicle.

Figure 4 shows both reference horizon and virtual horizon estimated by the proposed method for the highway traffic video clips. In the first clip where subject vehicle encounters stopped target vehicle, horizon error of the proposed method is very low as shown in Figure 4(a). Average and standard deviation of differences between reference horizon and virtual horizon are 1.4 pixels and 1.2 pixels, respectively. In the second and the third clips where subject vehicle follows moving target vehicle, horizon error is increased as shown in Figures 4(b) and 4(c). As both subject and target vehicles move, degraded accuracy of object detection results in increased error in these clips. Average and standard deviation of differences are 2.8 pixels and 1.9 pixels for the second clip, respectively, and 3.4 pixels and 2.0 pixels for the third clip, respectively. In the highway traffic video clips, there is only one vehicle ahead of subject vehicle as shown in Figure 3(a). As the proposed method estimates virtual horizon from average width and average position of vehicles, estimation accuracy can be improved, if accuracy of object detection is improved and several vehicles are detected in a frame.

**Figure 5:**
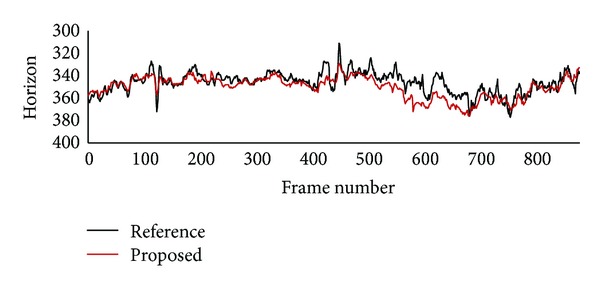
Reference horizon and virtual horizon estimated by the proposed method for urban traffic video clip.

Figure 5 shows both reference horizon and virtual horizon for the urban traffic video clip. Even though there are a lot of fluctuations on both reference and virtual horizons, their positions are quite similar in most frames. Average and standard deviation of differences between reference and virtual horizons are 6.0 pixels and 5.0 pixels, respectively. If we take into consideration that reference horizons vary in the range of 66 pixels in the urban traffic video clip, whereas they vary in the range of 12 pixels in the highway traffic video clips, it can be said that the accuracy is not degraded in the urban traffic video clip. Even though the clip includes a crossroad section and a hill section, accuracy is not degraded, since there are several vehicles in the clip as shown in Figure 3(b). This result confirms that the proposed method can be appropriately used in urban traffic environment.

**Figure 6:**
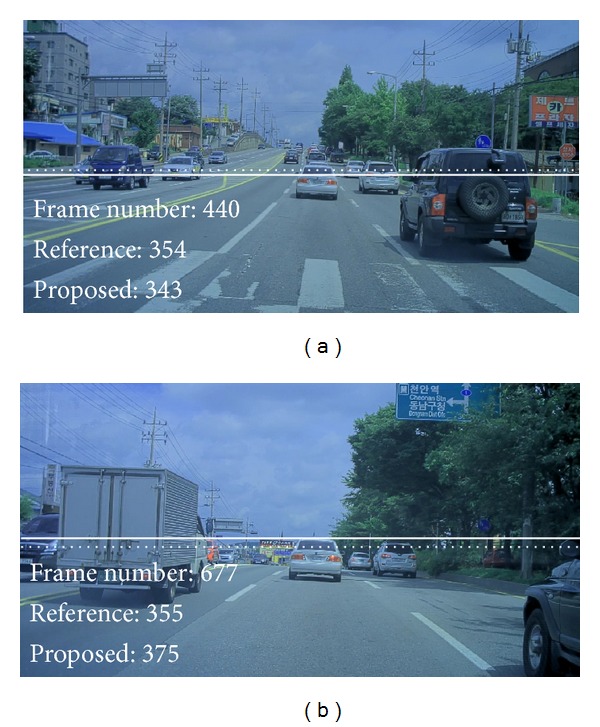
Reference horizon (solid line) and virtual horizon (dotted line) estimated by the proposed method for image frames from a hill section.

While subject vehicle goes over a hill between frame number 400 and frame number 700 in the urban traffic video clip, difference between reference horizon and virtual horizon increases in Figure 5. In a hill section where road inclination varies continuously, horizon located by analyzing lane markings in close proximity may be inappropriate to estimate range to a vehicle far away, since vehicles will appear above usual position in an uphill section and below in a hilltop section. On the other hand, virtual horizon estimated by the proposed method is appropriate to estimate range on hilly roads, since virtual horizon is estimated only from information on vehicle size and position.  Figure 6 shows both reference horizon and virtual horizon for image frames from hill section. Virtual horizons estimated by the proposed method are located above reference horizons in an uphill section image and below in a hilltop section image.

### 4.2. Evaluation of Range Estimation

**Figure 7:**
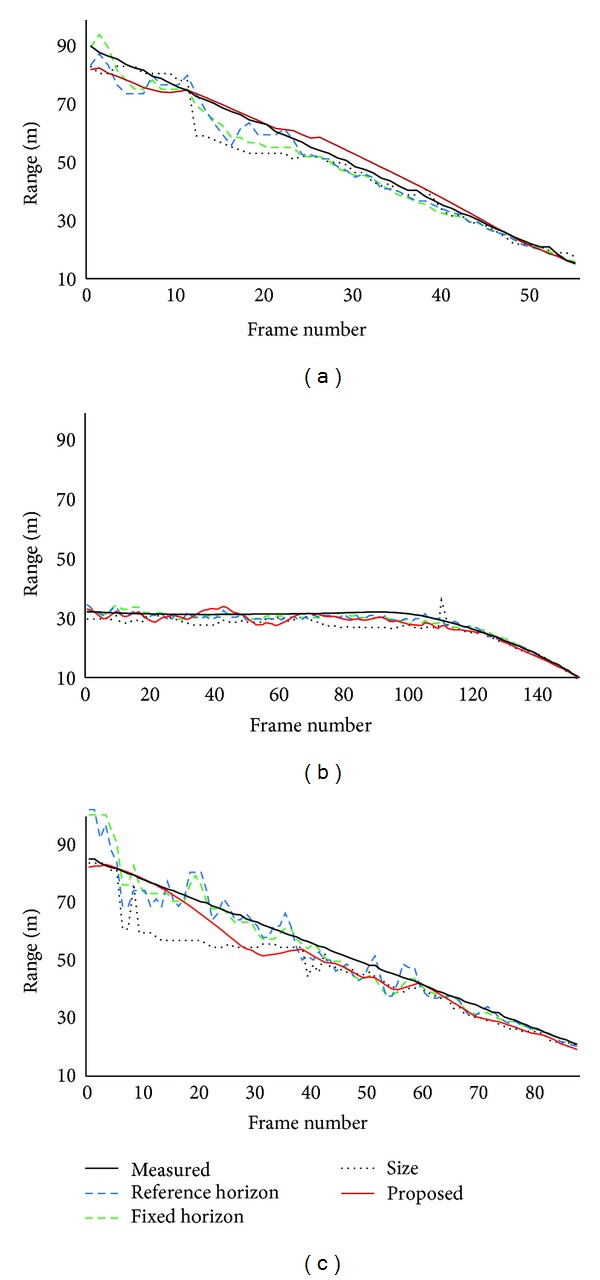
Measured and estimated ranges for highway traffic video clips. (a) Stopped target vehicle; (b) decelerating target vehicle; (c) slower moving target vehicle.

For the highway traffic video clips, ranges estimated by using the proposed method are compared with measured ranges and ranges estimated by using conventional methods.  Figure 7 shows measured and estimated ranges for each video clip. For all the video clips, each estimation method provides quite accurate range when target vehicle is within 50 m range. While target vehicle is out of 50 m range, each estimation method shows increased range error due to degraded accuracy of object detection in our FCW system. In each highway traffic video clip, there was only one vehicle ahead of subject vehicle. As mentioned in Section 4.1, range error may be reduced if several vehicles are detected in a frame.  Table 1 shows average and standard deviation of range error for each highway traffic video clip. Average range error of the proposed method is comparable to that of estimation with vehicle position information with reference horizons.

**Figure 8:**
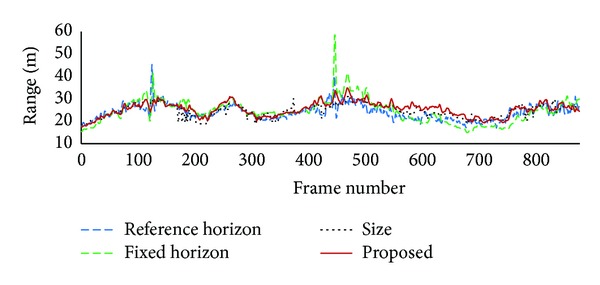
Estimated ranges for urban traffic video clip.

For the urban traffic video clip, measured ranges were not prepared. Ranges estimated by using the proposed method are compared with those estimated by using the conventional methods. In the previous experiment, estimated ranges were quite accurate when target vehicle is within 50 m range. To get more accurate range, the urban traffic video clip was recorded while subject vehicle was following a target vehicle with maintaining headway to the target vehicle at about 30 m.  Figure 8 shows estimated ranges for the urban traffic video clip. Ranges estimated from vehicle position with a fixed horizon show big fluctuations in some frames while those estimated by the other methods are very similar in most frames. This result shows that the estimated method from vehicle position with a fixed horizon cannot be used in urban traffic environment.

From frames 400 to 700 where target vehicle goes over a hill section, ranges estimated from vehicle position with reference horizons become smaller than those estimated from vehicle size, while those estimated by using the proposed method are similar to those estimated from vehicle size. Target vehicle appears in lower position than usual on a hilltop and ranges estimated from vehicle position become smaller. As ranges estimated from vehicle size are not influenced by vehicle position, they can be more accurate than those estimated from vehicle position in those frames. In the proposed method, lowered vehicle position is already compensated when virtual horizon is estimated, and range estimation will not be influenced by lowered vehicle position. Virtual horizons are located lower than reference horizons in those frames as shown in Figure 5. This result confirms that the proposed method is more appropriate while road inclination varies continuously on hilly roads.

## 5. Conclusion

In this paper, we propose a range estimation method which can be used for vision-based forward collision warning systems both in highway and urban traffic environments. The proposed method estimates virtual horizon from information on size and position of vehicles in image which is obtained by object detection algorithm and calculates range from vehicle position in image with the virtual horizon. In the conventional approach where horizons are determined by analyzing lane markings, horizons cannot be appropriately located when road inclination varies continuously on hilly roads or lane markings are not seen on crowded roads. On the other hand, virtual horizons are always located if vehicles are detected in image, as the proposed method estimates horizons only from information on size and position of vehicles in image. Small variation in position of horizon may result in large range error. Horizons determined by analyzing lane markings can be located even below vehicles, especially when the vehicles are on a hill, which can result in unbounded range error. Virtual horizons are always located above bottom lines of detected vehicles as they are estimated from vehicle positions, and range error is bounded. For experiments, a vision-based forward collision warning system has been implemented and the proposed method is evaluated with video clips recorded in highway and urban traffic environments. Experimental results confirm that the proposed method provides robust results in urban traffic environment as well as in highway traffic environment.

## Figures and Tables

**Table 1:** Average (**μ**) and standard deviation (**σ**) of range error for highway traffic video clips.

Test video clip	Reference horizon		Fixed horizon		Size		Proposed	
	*μ*	*σ*	*μ*	*σ*	*μ*	*σ*	*μ*	*σ*
Stopped target vehicle	5.3%	3.6%	5.8%	3.2%	7.2%	5.6%	4.6%	2.9%
Decelerating target vehicle	3.1%	2.2%	3.4%	2.4%	8.1%	4.7%	5.0%	3.2%
Slower moving target vehicle	6.7%	4.8%	5.4%	5.0%	11.2%	6.9%	7.1%	4.5%
